# Does Hypothermic Circulatory Arrest for Aortic Surgery Trigger Near-Death Experience? Incidence of Near-Death Experiences after Aortic Surgeries Performed under Hypothermic Circulatory Arrest

**DOI:** 10.1055/s-0041-1725091

**Published:** 2021-10-19

**Authors:** Marion Mauduit, Amedeo Anselmi, Jacques Tomasi, Reda Belhaj Soulami, Florent Le Bars, Erwan Flecher, Jean-Philippe Verhoye

**Affiliations:** 1 Department of Thoracic and Cardiovascular Surgery, Rennes University Hospital Center, Rennes, France

**Keywords:** near-death experience, circulatory arrest, hypothermia, aortic surgery

## Abstract

**Background** Understanding near-death experiences (NDE) could provide a new insight into the analysis of human consciousness and the neurocognitive processes happening upon the approach of death. With a temporary interruption of systemic perfusion, aortic surgery under hypothermic circulatory arrest (HCA) may be the only available model of reversible clinical death. We present, herein, the results of an observational study designed to assess the incidence of NDE after aortic surgery.

**Methods** We performed a prospective study including consecutive patients who underwent thoracic aortic surgery between July 2018 and September 2019 at our institution. Procedures without HCA were included to constitute a control group. The primary outcome was the incidence of NDE assessed with the Greyson NDE scale during the immediate postoperative course, via a standardized interview of the patients in the surgical ward.

**Results** One hundred and one patients were included. Twenty-one patients (20.8%) underwent nonelective interventions for aortic dissection. Ninety-one patients had hemiarch replacement (90.1%). Sixty-seven (66.3%) interventions were performed with HCA, with an average circulatory arrest duration of 26.9 ± 25.5 minutes, and a mean body temperature of 23.7 ± 3.8°C. None of the patients reported any recollection from their period of unconsciousness. There was no NDE experiencer in the study cohort.

**Conclusion** Several confounding factors regarding anesthesia, or NDE evaluation, might have impaired the chance of NDE recollections, and might have contributed to this negative result. Whether HCA may trigger NDE remains unknown.

## Introduction

Understanding the relationship between mind, consciousness, and the brain is perhaps one of the greatest challenges for the 21st century neuroscience. Even though the concept of human consciousness has been investigated by neuroscientists, psychologists, neurobiologists, and philosophers, its origin remains elusive. 1 The study of near-death experiences (NDE) could provide new prospects in the analysis of human consciousness and its relationship with the brain. 2 The occurrence of NDE when individuals are unconscious, such as those reported by cardiac arrest survivors, 3 4 raises questions about the real nature of consciousness, and the neurocognitive process happening upon the approach of death. Circulatory arrest is required to allow for a bloodless operative field, while hypothermia provides the brain and end-organ protection by significantly reducing the global metabolic demand. 5 6 With a temporary interruption of cerebral and systemic perfusion, aortic surgery under hypothermic circulatory arrest (HCA) may be the only available model of reversible clinical death.

Does the circulatory arrest performed during aortic surgery trigger NDE as does spontaneous cardiac arrest? To date, there is no published data regarding NDE and aortic surgery under HCA. We present, herein, the results of a prospective study designed to assess the incidence and characteristics of NDE after thoracic aortic surgery.

## Materials and Methods

### Study Design

We performed a prospective observational study including consecutive patients who underwent thoracic aortic surgery, with or without HCA, between July 2018 and September 2019 at our Institution. Patients who underwent aortic surgeries without HCA were included to create a control group. We obtained ethics committee approval and all patients gave informed consent.

### Surgical Technique

The aortic diseases affecting the patients were degenerative aneurysms or acute Type A aortic dissection. Surgeries were performed either electively, or on an urgent or emergent basis. Surgical procedures included aortic hemiarch or total arch replacement, combined with aortic root or aortic valve replacement when indicated. Operative strategy was guided by the nature and extent of the aortic disease, and HCA was performed when an open distal anastomosis was required.

After median sternotomy, cardiopulmonary bypass (CBP) was initiated via the axillary or the femoral artery, or through direct ascending aorta cannulation.

In case of HCA, systemic cooling was initiated on bypass. After achievement of the target body temperature, systemic perfusion was discontinued with the patient in the Trendelenburg position. The patient's head was packed in ice throughout the circulatory arrest to ensure topical cooling.

Cerebral protection was provided by moderate hypothermia (between 21 and 28°C, as defined by the current consensus). 7 Adjunctive antegrade cerebral perfusion (ACP) was used when the expected HCA duration exceeded 30 minutes. ACP was performed through either a bilateral perfusion strategy (such as by direct cannulation of the brachiocephalic artery and the left common carotid artery from inside the arch) or a unilateral perfusion strategy (cannulation of the right axillary artery with cross-clamp of the brachiocephalic artery) with a flow rate of 10 mL/kg/min. After completion of the aortic repair, the patients were gradually rewarmed (1°C/5 minutes, with a temperature gradient <10°C) and weaned from CPB.

### Anesthesia

A continuous intravenous (IV) lidocaine infusion (1 mg/kg/h) was started prior to incision after a bolus injection (1.5 mg/kg) and was continued until the end of the surgery. General anesthesia was induced with IV propofol (2–4 mg/mL) and remifentanil (2–6 ng/mL), administered with a target-controlled infusion system using the Schnider and Minto models, respectively. 8 Propofol target-control infusion was used for the maintenance of general anesthesia. Inhaled anesthetics were not used for maintenance. Low dose IV ketamine (0.10 mg/kg) was administered to prevent postoperative hyperalgesia. For neuromuscular block, cisatracurium (0.15 mg/kg) was used. In case of hemodynamic instability prior to induction, general anesthesia was induced using a combination of etomidate (0.3 mg/kg), sufentanil (0.15 µg/kg), and suxamethonium (1 mg/kg). General anesthesia was induced with etomidate instead of propofol in seven cases (6.9%).

Monitoring during anesthesia involved continuous electrocardiography, continuous pulse oximetry, invasive blood pressure monitoring, capnography, and blood gas and electrolyte measurement at regular intervals. The bispectral index was used to monitor the depth of anesthesia (target range: 40–60).

If HCA was performed, patients received 1,000 mg of thiopental, 1,000 mg of methylprednisolone, 3,000 mg of sulfate magnesium, and 250 mL of mannitol, prior to the circulatory arrest. Acid–base balance was managed with the α-stat method. Temperature monitoring was conducted with esophageal and bladder probes. Near-infrared spectroscopy was used to assess symmetry of cerebral perfusion. No anesthetic was provided during circulatory arrest.

Emergence from general anesthesia was allowed in the intensive care unit after at least 3 hours from the end of the surgical procedure, mainly to ensure hemodynamic stability and the absence of bleeding.

### Near-Death Experiences Evaluation

In the absence of hemodynamic instability or cognitive impairment, we performed a standardized interview of the patients in the surgical ward starting from postoperative day 4. Information about the study was delivered to the eligible patients only after the surgery, during the postoperative course. The interviews were conducted by a single physician. Patients were asked if they remembered the period of unconsciousness, and what they recalled. They were then given the Greyson NDE scale, a standardized 16-item multiple choice questionnaire used to discriminate patients who experienced NDE from those who did not. 9 The scale is divided into questions about cognitive processes, affective processes, purportedly paranormal elements, and transcendental elements. The Greyson NDE scale used in the study is indicated in Table 1 . This translation of the Greyson scale was used in previous studies conducted in French. 10

**Table 1:** The Greyson near-death experience scale

1. Did time seem to speed up or slow down? 0 = No 1 = Time seemed to go faster or slower than usual 2 = Everything seemed to be happening at once; or time stopped or lost all meaning
2. Were your thoughts speeded up? 0 = No 1 = Faster than usual 2 = Incredibly fast
3. Did scenes from your past come back to you? 0 = No 1 = I remembered many past events 2 = My past flashed before me, out of my control
4. Did you suddenly seem to understand everything? 0 = No 1 = Everything about myself or others 2 = Everything about the universe
5. Did you have a feeling of peace or pleasantness? 0 = No 1 = Relief or calmness 2 = Incredible peace or pleasantness
6. Did you have a feeling of joy? 0 = No 1 = Happiness 2 = Incredible joy
7. Did you feel a sense of harmony or unity with the universe? 0 = No 1 = I felt no longer in conflict with nature 2 = I felt united or one with the world
8. Did you see, or feel surrounded by, a brilliant light? 0 = No 1 = An unusually bright light 2 = A light clearly of mystical or other-worldly origin
9. Were your senses more vivid than usual? 0 = No 1 = More vivid than usual 2 = Incredibly more vivid
10. Did you seem to be aware of things going on elsewhere, as if by extrasensory perception? 0 = No 1 = Yes, but the facts have not been checked out 2 = Yes, and the facts have been checked out
11. Did scenes from the future come to you? 0 = No 1 = Scenes from my personal future 2 = Scenes from the world's future
12. Did you feel separated from your body? 0 = No 1 = I lost awareness of my body 2 = I clearly left my body and existed outside it
13. Did you seem to enter some other, unearthly world? 0 = No 1 = Some unfamiliar and strange place 2 = A clearly mystical or unearthly realm
14. Did you seem to encounter a mystical being or presence, or hear an unidentifiable voice? 0 = No 1 = I heard a voice I could not identify 2 = I encountered a definite being, or a voice clearly of mystical or unearthly origin
15. Did you see deceased or religious spirits? 0 = No 1 = I sensed their presence 2 = I actually saw them
16. Did you come to a border or point of no return? 0 = No 1 = I came to a definite conscious decision to “return” to life 2 = I came to a barrier that I was not permitted to cross or was “sent back” against my will

Note: Sum of all 16 items = total near-death experience scale score. A score of 7 or higher is considered as NDE.

### Definitions

Patients were divided into two groups: the “HCA group,” including patients who underwent HCA during the aortic reconstruction and the “control group,” including patients who underwent thoracic aortic procedures without HCA.

The primary outcome of interest was the incidence of NDE during aortic surgery. The secondary outcomes were the identification of risk factors for the occurrence of NDE, the characterization of NDE, and the occurrence of any out-of-body experience. The main postoperative outcomes, including 30-day mortality, stroke, and neurologic disturbance, are reported.

An NDE experiencer was defined as a patient who scored 7 or more on the Greyson NDE scale, according to the current consensus. 9 10

A 30-day mortality was defined as death occurring during the index hospitalization or within the 30th postoperative day if occurring after discharge. Stroke was defined as the onset of a new focal or global neurological deficit, or coma, with computed tomography or magnetic resonance imaging demonstration of a new ischemic/hemorrhagic lesion during the postoperative course. Temporary neurologic disturbance was defined as postoperative confusion, delirium, or seizure with normal cerebral imaging. Shock was defined as persistent hypotension requiring the administration of catecholamines to keep mean arterial blood pressure above 65 mm Hg

### Statistics

Continuous and categorical variables are presented as mean with standard deviation or median with ranges and frequencies with percentage, respectively. Statistical tests were not performed in this study.

## Results

### Patients and Operative Data

A total of 101 patients were consecutively included in this study. The mean age was 63.5 **±** 11.9 years and 67 (78.2%) were male. Detailed patients' characteristics are summarized in Table 2 . Nine patients had a psychiatric history and 14 had a preoperative neurological disorder. Twenty-one patients (20.8%) underwent nonelective interventions for aortic dissection.

**Table 2:** Preoperative conditions

Variables	Overall ( *n* = 101)		HCA group ( *n* = 67)		Control group ( *n* = 34)	
	*n*	%	*n*	%	*n*	%
*Demographic data* :						
Age (y) Mean ± SD	63.5 ± 11.9		63.7 ± 12.0		63.1 ± 11.9	
Male sex	67	78.2	49	73	30	88.2
*Comorbidities* :						
Neurologic disorder	14	13.8	7	10.4	7	20.6
Psychiatric disease	9	8.9	5	7.4	4	11.7
*Aortic pathology* :						
Acute Type A dissection	21	20.8	21	31.3	0	0
Elective surgery (aneurysm)	80	79.2	46	68.6	34	100

Abbreviations: HCA, hypothermic circulatory arrest; SD, standard deviation.

Ninety-one patients had hemiarch replacement (90.1%). Total arch replacement was required in 10 (9.9%) cases.

The average duration of surgery, myocardial ischemia time, and general anesthesia duration were 302.7 ± 101.6 minutes, 112.1 ± 39.2 minutes, and 14.4 ± 12.1 hours, respectively.

Thirty-four interventions were performed without HCA. For the 67 aortic procedures with HCA, the average circulatory arrest duration was 26.9 ± 25.5 minutes, with a mean body temperature of 23.7 ± 3.8°C. ACP was used in 31.3% ( *n* = 21) of the interventions with HCA (unilateral ACP in 6 and bilateral ACP in 15; Table 3 ).

**Table 3:** Operative data

Variables	Overall ( *n* = 101)		HCA group ( *n* = 67)		Control group ( *n* = 34)	
	*n*	%	*n*	%	*n*	%
*Aortic procedure* :						
Hemiarch replacement	91	90.1	57	85.1	34	100
Total arch replacement	10	9.9	10	14.9	0	0
*Times* :	Mean ± SD		Mean ± SD		Mean ± SD	
Surgical procedure (min)	302.7 ± 101.6		304.3 ± 88.4		299.3 ± 124.9	
Myocardial ischemia (min)	112.1 ± 39.2		111.5 ± 38.9		113.5 ± 40.4	
General anesthesia (h)	14.4 ± 12.1		15.7 ± 13.7		11.9 ± 7.8	
*Circulatory arrest details* :						
Arrest temperature (°C)	ND		23.7 ± 3.8		ND	
HCA time (min)	ND		26.9 ± 25.5		ND	
Procedures with ACP * n* (%)	ND		21	31.3	ND	

Abbreviations: ACP, antegrade cerebral perfusion; HCA, hypothermic circulatory arrest; ND, no data; SD, standard deviation.

During the postoperative course, 29.7% ( *n* = 30) of the patients received nonbenzodiazepine hypnotics for short-term treatment of insomnia (either zopiclone 7.5 mg or zolpidem 10 mg), 28.7% ( *n* = 29) of them received benzodiazepines (alprazolam 0.25–0.50 mg or bromazepam 1.5–6 mg). Both nonbenzodiazepine hypnotics and benzodiazepines were administered in 8.6% of cases ( *n* = 9).

### Postoperative Outcomes

There was no death at 30 days. The postoperative stroke rate was 1.9% ( *n* = 2). Temporary neurologic disturbance was described in 15 (14.8%) patients. Early reintervention for bleeding was required in 16 (15.8%) cases. The mean intensive care unit stay was 3.4 days. The postoperative outcomes are summarized in Table 4 .

**Table 4:** Postoperative outcomes

Variables	Overall ( *n* = 101)		HCA group ( *n* = 67)		Control group ( *n* = 34)	
	*n*	%	*n*	%	*n*	%
30-day mortality	0	0	0		0	0
Stroke	2	1.9	1	1.5	1	2.9
Seizure	3	2.9	3	4.5	0	0
Confusion	12	11.9	9	13.4	3	8.8
*Reintervention for bleeding* :	16	15.8	13	19.4	3	8.8
Shock	3	2.9	1	1.5	2	5.9
ICU duration (d) Mean ± SD	3.4 ± 2.3		3.5 ± 2.2		3.3 ± 2.6	

Abbreviations: HCA, hypothermic circulatory arrest; ICU, intensive care unit; SD, standard deviation.

### Near-Death Experiences Evaluation

The interviews were performed in the surgical ward between postoperative day 4 and day 9 (5.5 ± 2.3 days; median, 5 days) and lasted 15 minutes, on average. None of the patients reported any recollection of their period of unconsciousness. There was no NDE experiencer in the study cohort.

## Discussion

NDE are subjective experiences involving the memory of impressions during a particular state of consciousness, including pleasant feelings, acceleration or slowing of time, the sight of a tunnel or a light, the sensation of reliving elements of the past, or seeing deceased relatives. 4 11 Ring 12 identified a common framework for NDE through structured interviews of NDE experiencers, and described five successive stages: feeling of peace and wellbeing, separation of the body, entering a “tunnel-like” dark area, seeing a bright light, and entering the light. To identify NDE, Ring first developed the “weighted core experience index,” which was further refined by Greyson, 9 arguing that the components of Ring's scale lacked specificity. 3 The Greyson NDE scale has demonstrated good reliability and internal consistency and is commonly used in the literature. 13

Three major theories have been suggested in an attempt to explain the occurrence of NDE as follows: (1) the physiological changes that accompany the death process, (2) an adaptive dissociative mechanism in response to the death threat, or (3) the onset of a transcendental experience. 3 Several physiological factors have been considered as potential mediators of NDE, such as cerebral hypoxia, hypercarbia, endorphins, ketamine and N-methyl-D-aspartate (NMDA) receptors, serotonin signaling pathways, limbal system activation, or anoxic seizures of the temporal lobe. 14 15 16 Despite these theoretical approaches, the origin of NDE remains elusive for the scientific community.

The majority of NDE experiencers are found among cardiac arrest survivors. Prospective studies among this specific population report an incidence ranging from 6 to 12% of NDE. 4 Data from animal studies demonstrated an isoelectric electroencephalogram (EEG) within 10 to 20 seconds from the onset of the cardiac arrest. 17 The description of NDE during circulatory arrest with structured thoughts, feelings, emotions, and self-awareness, even though there is no indicator of brain activity, raises many questions about the nature of human consciousness and the relationship between the brain and consciousness. 18

Aortic surgery with HCA was thought to be a potential reversible model of “clinical death,” which could provide insight into the potential process happening upon the approach of death. During HCA, patients fulfil the criteria of clinical death, with no brain perfusion, and no spontaneous or artificial ventilation. 19 Indeed, hypothermia reduces tissue oxygen and metabolic demand to the extent that systemic perfusion may be interrupted for limited periods. 20

To differentiate the confounding effects of general anesthesia from the consequence of circulatory arrest, patients who had thoracic aortic surgery both with and without HCA were included in our study. In the scientific literature, few cases of NDE during general anesthesia are reported and they may be distinguished from “intraoperative awareness.” 21 Awareness refers to a recall of real facts that actually happened during anesthesia and may be related to inadequate narcosis, contrary to NDE, which is about a subjective experience.

In our cohort, no NDE was reported by the patients. Several factors might explain this negative result. First of all, the hypnotic agents and analgesics used during general anesthesia may induce retrograde amnesia, or merely prevent NDE, although some NDE during anesthesia have previously been described. 21 The potential influence of modified neurotransmitter release and systemic inflammatory response induced by the CPB, along with the varying degrees of ischemia/reperfusion during aortic surgery, should also be taken into consideration. 22 The duration of unconsciousness in our study (14.4 hours on average), inherent to the prolonged general anesthesia, might prevent the patient from remembering NDE events. Furthermore, the time between awakening from anesthesia and the patient's interviews might have been too long, though in the study by van Lommel et al, 4 NDE memories did not fade out with time, as most NDE experiencers were able to retell accurately their experience 2 years later. The number of patients included in our study might also be too limited to evidence NDE, although the incidence rates reported among cardiac arrest survivors suggest that such cohort size should be adequate. 3

The level of hypothermia and the optional use of adjunctive cerebral perfusion during circulatory arrest might also play a crucial part. Keenan et al 23 monitored EEG during 71 aortic arch replacement with HCA and ACP. They highlighted an immediate loss of electrocerebral activity after circulatory arrest in 45% of the patients which was rapidly restored with the establishment of selective cerebral perfusion, except for two patients. Nonetheless, the core temperature in our study was below the threshold used in their cohort (27.8 vs. 23.7°C). Indeed, a continuous EEG pattern before the initiation of circulatory arrest was observed in 66% of the patients, while 34% presented a burst suppression pattern. Stecker and colleagues 24 described the neurophysiologic changes during cooling before circulatory arrest and found that EEG burst suppression appeared at 24.4 ± 4°C, and electrocerebral silence at 17.8 ± 4°C. It should be kept in mind that although NDE are supposed to be experienced “during a period of clinical death with flat EEG,” an isoelectric EEG might not be a necessary condition for the onset of NDE. Besides, the absence of NDE in our cohort precludes any analyses about the potential impact of ACP during HCA. EEG was also not monitored in our study. Furthermore, hypothermia reduces the release of excitatory neurotransmitters such as glutamate. 25

## Conclusion

No patients reported NDE after thoracic aortic surgery, with or without HCA. Several confounding factors regarding anesthesia, or NDE evaluation, might have impaired the chance of NDE recollection. Thus, whether HCA may trigger NDE remains unknown. Nevertheless, the field of cardiothoracic surgery, with its inherent control over cerebral and systemic perfusion, might still be considered for a study model to grasp some aspect of the complex reality of death.

## Notes

Editor's Commentary I suspect that many of us have wondered what our patients experience during deep hypothermic circulatory arrest (DHCA). I have applied this technique thousands of times, all without any cerebral perfusion (i.e., “straight” DHCA), and I have often contemplated what the experience may be like from the patient's point of view. During my training, decades ago, cardiac arrest and resuscitation were quite common after cardiac surgery. I remember a patient waking up intermittently, and looking at me and talking to me, as I massaged her heart after reopening her sternotomy. Patients often did report near-death experiences (NDEs) after cardiopulmonary resuscitation. But no patient has reported to me an NDE after DHCA. I am especially interested in this topic, because I myself experienced an NDE. When I was 20 years old, my classmate and I were driving in two different cars from Yale University to Vassar College, as we did most weekends. We often made those weekend trips in the days before the Ivy League schools became coeducational. It was a fall New England day, and the temperature was in the low 30's (Fahrenheit) --near freezing. My classmate was driving a 1988 Mustang GT500 Shelby Cobra 428, and I was in my 1967 Corvette Stingray 327. We often drove very fast, essentially racing each other on public roads. It began to rain. We were on a small, winding, two-lane country road. There was a narrow bridge just up ahead. My classmate was ahead of me. I saw his car start to fishtail as he entered onto the bridge. I braked my car, surmising that the light rain was freezing on the bridge surface. I wanted to slow my speed, to avoid any issue on the bridge. Instead, my car also fishtailed, and within a moment, it was completely out of control on the narrow, icy bridge. In fact, it was traveling directly backwards toward the bridge. I had no control of the car whatsoever. I remember this event as if it was yesterday. My first thought, in real time, was that this beautiful fiberglass Corvette would be shattered. Right after that thought, I realized I was about to die. There was no doubt at all in my mind. In the next few moments, I literally saw my whole life (all 20 years) flash before my eyes. It seemed that I re-lived every moment at warp speed. Ultimately, the car did not crash. It stopped on its own, parallel parking perfectly only inches from the feeble wall of the bridge --without a scratch. I got out of the car with the help of my classmate. I was shaking. I could not stand. My knees could not hold me. Eventually, I regained some composure and we continued our road trip --a bit more slowly. This memory is absolutely etched in my mind, even now, many decades after its occurrence. NDE is often ascribed to brain hypoperfusion due to hemodynamic abnormalities. My experience was devoid of any physical abnormalities. My body was physically fine. Rather, this was solely an emotional experience, inducing a powerful NDE. This personal experience, along with my having employed DHCA thousands of times, had always made me wonder if patients had any NDE-type sensations related to their surgery under DHCA. The article by Mauduit and colleagues takes a careful, precise, thorough look at this issue. They find no evidence of NDE after DHCA.
